# Ischemia/reperfusion activates myocardial innate immune response: the key role of the toll-like receptor

**DOI:** 10.3389/fphys.2014.00496

**Published:** 2014-12-18

**Authors:** Gemma Vilahur, Lina Badimon

**Affiliations:** ^1^Cardiovascular Research Center, CSIC-ICCC, Hospital de la Santa Creu i Sant Pau, Institut d'Investigació Biomèdica Sant PauBarcelona, Spain; ^2^Cardiovascular Research Chair, Universitat Autònoma de BarcelonaBarcelona, Spain

**Keywords:** myocardium, ischemia/reperfusion injury, innate immune response, toll-like receptor, cytokines, inflammation

## Abstract

Recent data have indicated that the myocardium may act as an immune organ initiating cardiac innate immune response and inflammation. It has been suggested that activation of the immune system occurs upon the interaction of damage-associated molecular patterns (DAMPs) generated and released during ischemic damage with pattern recognition receptors (Toll like receptors; TLR) present in cardiac cells. Among TLRs, TLR4, and TLR2 are the ones mostly expressed in cardiac tissue. Whereas TLR4 has shown to play a detrimental role in myocardial ischemia/reperfusion (I/R) injury, the effect elicited by TLR2 activation remains controversial. Once activated, TLR signaling may occur via the Myd88- and Trif- dependent pathways leading to NFκB and IFN-3 activation, respectively, and subsequent stimulation of pro-inflammatory and immunomodulatory cytokine gene expression. Cytokine release contributes to neutrophils activation, recruitment, adhesion and infiltration to the site of cardiac injury further perpetuating the inflammatory process. This mini-review will focus on the current knowledge regarding the role of the heart in inducing and coordinating the innate inflammatory response via the TLR signaling pathway in myocardial I/R injury.

## Activation of myocardial TLR in ischemia/reperfusion

**Figure 1:**
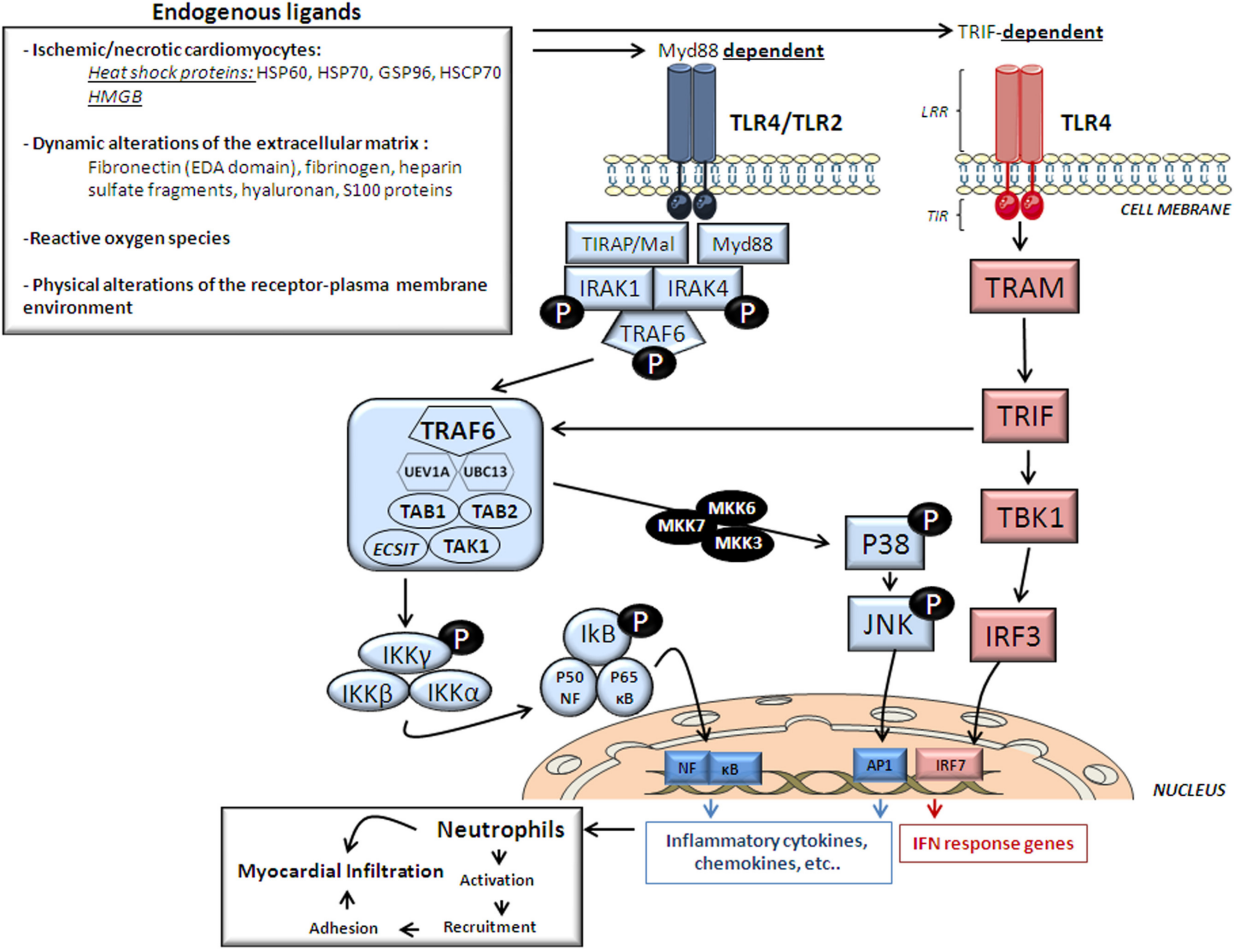
**Cardiac TLR2/4 structure, endogenous ligands and signaling**. This figure depicts the endogenous ligands released upon ischemia and reperfusion that trigger signaling pathways activated by the two most important cardiac TLR receptors, TLR2 and TLR4 and TLR structure. HMGB, high mobility group box; HSP, heat-shock protein; HSCP, heat shock cognate protein. LRR, extracellular C-terminal leucine-rich repeat (ligand recognition domain); TIR-domain, N-terminal cytoplasmic toll/interleukin receptor homologous signaling domain; IRAK, interleukin -1 receptor-associated kinase; TRAF6, tumor necrosis factor-receptor-associated factor-6; TAK1, TGF-β-Activated Kinase-1; TAB1, TAK1-Binding Protein-1; TAB2, TAK1-Binding Protein-2; UBC13, ubiquitin-conjugating enzyme-13; UEV1A, ubiquitin-conjugating enzyme E2-variant-1; IKK, Inhibitor of κ Light Polypeptide Gene Enhancer in B-Cells Kinase; NF-κB, nuclear transcription factor- κB; MKK, mitogen-activated protein kinase; TRIF, TIR domain-containing adaptor protein inducing type 1 interferons; TRAM, Trif-related adaptor molecule; TBK1, TANK Binding Kinase-1; IRF, interferon regulatory factor. AP-1, transcription factor complex-1.

Activation of the innate immunity is an essential component of the acute inflammatory response in the setting of ischemia/reperfusion (I/R). It is widely recognized the key role of neutrophils, monocytes, macrophages and dendritic cells in innate immunity. However, recent experimental lines of evidence have suggested that ischemic cardiomyocytes can also contribute to the innate immune response besides being a target organ. Activation of the immune response mainly occurs when “danger” signals released from the damaged cardiomyocytes [the so called damage-associated molecular patterns (DAMPs)] interact with pattern recognition receptors (PRRs). Among PRRs, Toll-Like Receptors (TLR), originally identified 20 years ago for its role in the embryonic development of the fruit fly *Drosophila melanogaster* (Anderson et al., 1985), have been later recognized as a key elements of the immune system (Lemaitre et al., 1996). Ten TLR have been identified so far in humans six of which are located in the cell surface and are activated by a variety of exogenous/endogenous ligands (TLR1, TLR 2, TLR4, TLR5, TLR6, and TLR10) and four are expressed intracellularly (TLR3, TLR7, TLR8, and TLR9) and are generally recognized by nucleic acid structures (Wang et al., 2010; Mann, 2011). Almost all cells of the heart have shown to express TLR (TLR 2, 3, 4, and 6 are found in cardiomyocytes, and TLR1 through 6 are found in the smooth muscle and endothelial cells) (Frantz et al., 2007). The discovery of the notably contribution of TLR2 and TLR4 in the development of atherosclerotic lesions and vascular remodeling (Kiechl et al., 2002; Lin et al., 2009) and their abundance in adult human hearts (Lin and Knowlton, 2014) frosted the interest in deciphering their potential role in the setting of myocardial I/R (Frantz et al., 1999). Although the mechanisms involved in TLR 2 and 4 activation in myocardial I/R remain to be fully described, a variety of endogenous molecules have been identified to act as potential endogenous DAMPS. In one hand, myocardial cell necrosis triggered during ischemia results in the release of necrotic products and cellular constituents such as heat shock proteins (HSP) and nuclear DNA-binding protein high mobility group box (HMGB-1) capable of activating the TLR2 and TLR4 signaling. Dynamic alterations of extra cellular matrix (ECM) resulting from I/R have also shown to generate DAMPs molecules. In this regard, either breakdown products of the cardiac ECM or *de novo* synthesized matrix molecules may function as endogenous ligands (Lin and Knowlton, 2014). Reactive oxygen species have also demonstrated to activate cytokine production through TLR4-dependent mechanisms, although the molecules involved remain unknown (Flohe et al., 1997; Ha et al., 2011). Finally, physical alterations of the receptor-plasma membrane environment are thought to activate TLRs in the absence of any cellular-/ECM-derived ligand (Figure 1) (Scaffidi et al., 2002; Scheibner et al., 2006). Once activated, signal transduction of TLR2 and/or 4 may take place via two main pathways, the Myd88- and TRIF- dependent pathway, eventually leading to cytokine production.

## TLR signal transduction

TLR are type-I transmembrane proteins composed of 3 structural domains: an extracellular C-terminal leucine-rich repeat (LRR) as ligand recognition domain, a central transmembrane domain, and a cytoplasmic domain similar to the IL-1 receptor (the toll/IL-1 receptor; TIR). Whereas TLR4 acts as homodimer, TLR2 forms heterodimers with TLR1 and TLR6 (Wang et al., 2010). TLR dimerization leads to changes in the conformation of the cytoplasmic TIR domain which subsequently facilitates the recruitment of adaptors that initiate TLRs downstream signaling. Depending on the usage of different adaptor molecules, TLRs signaling if divided in two major pathways, a MyD88-dependent pathway and a TRIF-dependent pathway.

### MyD88-dependent pathway

Myd88 adaptor protein requires the recruitment of a TIR domain-containing adaptor protein (TIRAP/Mal) to initiate TLR4/2-related signaling. As shown in Figure 1 once stimulated, the MyD88/TIRAP complex recruits and activates the downstream interleukin -1 receptor-associated kinase 4 (IRAK4) which induces IRAK1 phosphorylation and in turn recruits TNF-Receptor-Associated Factor-6 (TRAF6) by associating with phosphorylated IRAK1. Hereafter, phosphorylated TRAF6 then dissociates from the receptor and forms a complex with the linker molecules transforming growth factor β-activated kinase (TAK)-1, TAK1-Binding Protein-1 (TAB1) and TAB2. TRAF6, TAK1, TAB1, and TAB2 associate with the Ubiquitin ligases UBC13 (Ubiquitin-Conjugating Enzyme-13) and UEV1A (Ubiquitin-conjugating Enzyme E2-Variant-1). TAK1 is then activated and phosphorylates the IKK complex (Inhibitor of κ Light Polypeptide Gene Enhancer in B-Cells Kinase; IKK-α, IKK-β, and IKK-γ) and p38 Kinases by activating MKK3 (Mitogen-Activated Protein Kinase Kinase-3), MKK6 and MKK7. The IKK complex then phosphorylates I-κB which leads to its ubiquitylation and subsequent degradation. This allows NF-κB to translocate to the nucleus which brings about the production of pro-inflammatory cytokines and expression of co-stimulatory molecules (Ha et al., 2011). On the other hand, p38 Kinases activates c-Jun Kinase (JNKs) which then enter the nucleus and activates the transcription factor complex (AP1) which also stimulates the expression of pro-inflammatory cytokine genes (Figure 1) (Chao, 2009).

### TRIF-dependent pathway

TLR4 can also signal through a TRIF-dependent pathway (also known as the MyD88-independent pathway; Figure 1). In the TRIF-dependent pathway TLR4 may signal via TRIF-related adaptor molecule (TRAM) and TRIF which activates TBK1 (TANK Binding Kinase-1) eventually activating interferon-regulatory factor-3 (IRF3). This pathway may also signal through NF-κB signaling through TRAF6 recruitment. Activation of the TRIF-dependent pathway induces IFNβ and IFN-inducible genes including a variety of cytokines (Figure 1) (Chao, 2009).

All TLRs exert their action through the Myd88-dependent pathway except for TLR3 and partially for TLR4 which exert their action through the TRIF-dependent pathway. In fact, TRIF-knock-out mice has shown impaired cytokine production despite IRAK-1 phosphorylation and NF-κB activation suggesting that TLR4 requires both Myd88- and TRIF-dependent pathways to exert its action.

## Myocardial TLR4 a new player in I/R injury

Oyama et al. were the first to report a detrimental role for TLR4 in myocardial I/R injury (Oyama et al., 2004). Since then, several investigations have corroborated that TLR4 deficient mice show suppressed inflammatory response (lower NF-κB activation and reduced TNFα and IL1β levels), decreased cardiac inflammatory infiltration and smaller infarctions as compared with their wild-type littermates (Chong et al., 2004; Oyama et al., 2004). Moreover, administration of a specific TLR4 antagonist has shown to decrease NF-κB activation and subsequent pro-inflammatory cytokine release limiting the size of infarction as compared to non-treated animals (Kim et al., 2007a). However, inconsistent results have been reported regarding the role of TLR4 in cardiac recovery after I/R, some studies showing improvement (Kim et al., 2007b) and others no effect (Fallach et al., 2010).

A short-period of coronary ischemia (5 min) has not shown to trigger an inflammatory reaction (Bolli and Marban, 1999). On the contrary, brief ischemic periods implemented before an ischemic insult have shown to confer protection against prolonged ischemia (i.e., ischemic pre-conditioning) (Murry et al., 1986). However, sustained ischemia has shown to result in a detrimental inflammatory response. In this regard, we have reported in a preclinical model of closed-chest coronary balloon occlusion that 30 min of ischemia suffice to enhance the inflammatory potential of circulating leukocytes and increase cytokines release (Vilahur et al., 2009). Interestingly, such systemic inflammatory response was associated with a marked upregulation of both myocardial TLR-4- MyD88 -dependent and -TRIF-dependent signaling pathways in the ischemic myocardium leading to NF-κB nucleus translocation and IRF3 activation, respectively (Vilahur et al., 2011). These observations support a relatively prompt activation of the cardiac innate immune response upon an ischemic insult that rapidly translates into a pro-inflammatory systemic response through TLR4/cytokine networks. In addition, these data also suggests that timely interception of TLR4 might be critical to prevent adverse myocardial remodeling post-I/R (Vilahur et al., 2011). Once activated, myocardial TLR4-related inflammatory response stimulates the homing and recruitment of neutrophils to the site of cardiac injury (Oyama et al., 2004). Neutrophils recruitment and further infiltration may then contribute to the detected raise in cardiac TLR-4 expression detected up to 1 week after reperfusion (Vilahur et al., 2011). Although the relative role of neutrophils TLR4 and myocardial TLR4 in I/R-induced neutrophils cardiac infiltration has yet to be clearly defined, an interesting study in *Langendorff* perfused heart supported that functional TLR4 in the myocardium is required for neutrophils infiltration after global I/R. Besides, it has been suggested that myocardial TLR4 mediates cardiac chemokine response through a mechanisms involving HSC70, released during ischemia (Ao et al., 2009). Neutrophils are the major leukocytes found in I/R injury and not only are target cells for the cytokines released by the ischemic-reperfused myocardium but also contribute to the progression of I/R tissue damage. Released pro-inflammatory cytokines from the ischemic reperfused myocardium activates both neutrophils and cardiomyocytes leading to MAC-1 and intercellular adhesion molecule-1 (ICAM1) expression, respectively. MAC-1/ICAM1 interaction favors neutrophils adherence to cardiomyocytes which, in turn, accelerates and perpetuates tissue injury via reactive oxygen species (ROS) generation and pro-inflammatory cytokine release by both cell types triggering a positive feedback loop (Arslan et al., 2008).

## The controversial role of TLR2 in myocardial I/R injury

The role of myocardial TLR2 in the setting of I/R has been poorly investigated. In fact, it still remains uncertain whether it has a detrimental role in I/R injury similar to that of TLR4 or exerts beneficial effects. Shishido et al. (2003) reported that infarct size and the degree of inflammatory cell infiltration in the infarcted area were similar between wild type and TLR-2 knock-out mice. However, longer time-period analysis (up to 4 weeks) revealed that TLR-2 deficient mice showed lower fibrosis in the non-infarcted myocardium and improved left-ventricular remodeling parameters, as assessed by echocardiography, suggesting a key role for TLR2 in the tissue repair process after reperfusion (i.e., remodeling phase). Yet, it is unknown the relative contribution of TLR2 expressing inflammatory cells and myocardial-related TLR2 in myocardial I/R injury. On the other hand, studies in isolated hearts and infarcted mice have shown, by using specific synthetic TLR2 ligands, that TLR2 is required for ischemic pre-conditioning-induced left ventricle functional improvement and infarct size reduction and cardiac recovery, respectively (Dong et al., 2010; Mersmann et al., 2010).

## Concluding remarks

Innate immunity triggers the initial inflammatory response upon myocardial ischemia in part by the TLR signaling. Several lines of evidence suggest the active involvement of the heart in inducing and regulating the innate inflammatory response in the progression of myocardial I/R injury. Cardiac myocytes exhibit properties associated with the innate immune response, they are able to generate DAMPs and express TLRs. Although the mechanisms by which TLRs exert their detrimental cardiovascular effects in the setting of I/R are incompletely understood, during the last years emerging evidence has indicated that cardiac TLR4 contribute to cytokine release and neutrophils activation and recruitment to the site of injury (Aikawa et al., 2001). In contrast, the role of TLR2 still remains unclear. Further research is needed to explore the pathways and mechanisms by which the cardiac innate response is activated following tissue injury. Development of selective compounds capable to inhibit the TLR signaling may provide important insights in this regard. Yet, it is important to consider that although excessive pro-inflammatory cytokine production exerts myocardial detrimental effects in the ischemic reperfused myocardium, cytokines also drive the tissue repair process within the heart.

### Conflict of interest statement

The authors declare that the research was conducted in the absence of any commercial or financial relationships that could be construed as a potential conflict of interest.
